# An Account of Two Cases of Retroverted Uterus

**Published:** 1790

**Authors:** Richard Croft

**Affiliations:** Surgeon in London


XI. *An Account of two Caſes of retroverted Uterus. By Mr.* Richard Croft, *Surgeon in London.*

ALTHOUGH the retroverſion of the uterus is a ſubject which has of late years excited excited a great deal of attention among practitioners of midwifery, yet it is perhaps not yet generally known or allowed that the suppression of urine which occurs in these cases, and which has hitherto been supposed to be the consequence of the retroversion, ought rather to be considered as the cause than the effect of the latter affection. This theory * of the complaint, which enables us to account so satisfactorily for the manner in which it comes on, as well as for the means of relieving it, by drawing off the water, seems to derive farther confirmation from the following cases, (particularly from the first of the two) which is my reason for wishing to offer them to the Public.

### CASE I.

In the month of December, 1787, I was desired to visit Elizabeth Harrison, who had been near a month ill of a complaint, which was at first considered as a suppression of urine; but her extremities and abdomen having swelled considerably, and a fluctuation in the abdomen becoming evident, a surgeon to whom she ap-

* See Dr. Denman's Introduction to the Practice of Midwifery, Vol. I. page 130.

plied, plied, thinking ſhe had a dropſy, propoſed to tap her; and I was called to give my opinion of the propriety of the operation.

Every part of the patient's body, even her face, was then œdematous to a very conſiderable degree, and the abdomen was prodigiouſly large and painful.

From the account I received from the patient of the circumſtances which had occurred from the beginning of her illneſs, eſpecially of the manner in which the retention of urine had at firſt taken place, (which ſhe well recollected to have happened when ſhe was baulked of an opportunity of making water); from the occaſional flowing of a ſmall quantity of urine involuntarily; and from her being in the third month of her pregnancy, I deemed it neceſſary to examine her *per vaginam*. In that examination I found the fundus uteri preſſed very low between the vagina and rectum, and that the os uteri was drawn up beyond my reach. There were alſo the other ſymptoms which attend the retroverſion of the uterus.

The nature of the complaint being thus clearly aſcertained, I introduced a catheter, and drew off more than ſeven quarts of urine, by which ſhe was inſtantly relieved of a great

ſhare

ſhare of her pain, and the abdomen was nearly reduced to its proper ſize.

After the injection of a common purging clyſter, an opiate was adminiſtered. In ſix or eight hours from the time of uſing the catheter the abdomen was again very much diſtended, and the pain returned with violence; but as ſhe lived at a conſiderable diſtance from me, I could not ſee her for near fourteen hours, when I again drew off full ſix quarts of urine. She was then deſired to confine herſelf to an horizontal poſition, and had her urine drawn off three times in the courſe of every twenty-four hours for ſeveral days, and occaſionally for near three weeks; at the end of which time the fundus uteri had gradually riſen higher, and I could feel the os uteri deſcending into the pelvis. The tumefaction of the extremities ſoon diſappeared, and ſhe recovered the power of making water, which ſhe was cautioned to do very frequently. She went through the remainder of her pregnancy without any interruption, was delivered of a healthy child in May, 1788, after an eaſy labour, and recovered in the moſt favourable manner.

### CASE II.

In the beginning of laſt October I was called in haſte to ſee a poor woman in Davies Street, who was thought to be in the moſt imminent danger. She complained of excruciating pain in the abdomen, which was much ſwelled, and ſhe had not been able to void any urine for three days. She was in the third month of her pregnancy. On examining her *per vaginam* I readily perceived the fundus uteri preſſed very low down between the vagina and rectum, but the os uteri was out of my reach. I immediately introduced a catheter, and drew off a large quantity of urine, by which ſhe was directly made eaſy. I continued to uſe the catheter twice every day for ſix days, when the uterus ſuddenly recovered its proper poſition, and ſhe was able to make water. She has ſince gone on in her pregnancy without any complaint.

*Percy Street,*

December 4, 1790.

